# Predictors of Physical Abuse in Elder Patients With Fracture

**DOI:** 10.5435/JAAOSGlobal-D-22-00144

**Published:** 2022-07-12

**Authors:** Mursal Gardezi, Harold G. Moore, Lee E. Rubin, Jonathan N. Grauer

**Affiliations:** From the Yale School of Medicine, New Haven, CT (Gardezi); the Weill Cornell Medical College, New York, NY (Dr. Moore); and the Department of Orthopaedics and Rehabilitation, Yale New Haven Hospital, New Haven, CT (Dr. Rubin and Dr. Grauer).

## Abstract

**Introduction:**

Elder abuse is a public health issue requiring attention. Unlike abuse in the pediatric population, predictors of elder abuse in patients with fracture have not been well defined.

**Methods:**

Elderly patients with physical abuse and fracture were abstracted using the 2007 to 2017 National Emergency Department Sample database. Univariate comparisons, multivariate regression, and adjusted odds ratios were used to determine independent predictors of elder abuse compared with nonabuse fracture controls.

**Results:**

Thirteen percent of elder physical abuse patients presenting to the emergency department had fracture. Of all patients with fracture, elder abuse patients tended to be younger; be female; belong to lower income quartiles; and have codiagnoses of volume depletion, mental disorders, dementia, and intellectual disability. Presentation with other forms of elder abuse, such as psychological abuse, neglect, and sexual abuse, and multiple fractures were also associated with elder physical abuse. Multivariate regression found elder abuse to be more likely in the setting of skull and rib fractures and less likely in the setting of femur and foot and ankle fractures.

**Discussion:**

This study identified predictors of elder physical abuse in fracture patients older than 60 years. As with pediatric abuse, heightened awareness of potential physical abuse should be considered, especially in higher risk patients.

Elder abuse, which has an estimated lifetime incidence as high as 21%,^1^ is a pervasive issue in society with a pooled prevalence of nearly 16% in community settings^2^ and 33% in rural areas.^3^ Elder abuse can take the form of physical abuse, neglect, emotional/psychological abuse, sexual abuse, and/or financial exploitation.^4^ Such forms of abuse are associated with poor short-term^5,6^ and long-term health consequences,^6,7,8,9^ increased healthcare utilization,^6,10^ less social support,^1,5,6^ and increased mortality.^6,9,11,12^ As orthopaedic sequalae may be the presenting symptoms of such abuse, presenting orthopaedic patterns need definition.

For elder physical abuse, concern may be raised in the setting of delayed presentation or inconsistencies in the stated mechanism of injury.^13^ Previous studies evaluating fracture patterns correlating with elder abuse have been limited to single emergency department (ED) studies and have found fractures of the hand,^14^ face, and head^14,15^ to potentially correlate with abuse. One of these studies reported 46% of their population of individuals older than 65 years with fracture (total n = 652) had one or more potential correlates of abuse (although actual abuse was not confirmed).^15^ Overall, predictors of elder abuse in those presenting with fracture have not been as well defined based on more broad-reaching, national evaluations and may have been limited by patient numbers.

On the other end of the age spectrum, orthopaedic manifestations of child abuse are well characterized, although still likely underdiagnosed.^16-18^ Along a similar vein, intimate partner violence has been associated with orbital fractures.^19,20^ Analogous to the identification of fracture presentations characteristically associated with child and intimate partner abuse, this study aims to identify presenting patterns seen in association with elder abuse using a large national database.

## Methods

### Study Population

The National Emergency Department Sample is an all-payer US ED database that captures deidentified data for over 30 million ED visits per year from hospitals in the Healthcare Cost and Utilization Project. Exact values when observations were equal to or less than 10 are reported nonspecifically as per the Healthcare Cost and Utilization Project data usage agreement (throughout the article, this is represented with an “^a^”). Because this database contains only deidentified data, our institution's Investigation Review Board found studies using this database exempt from review.

**Figure 1:**
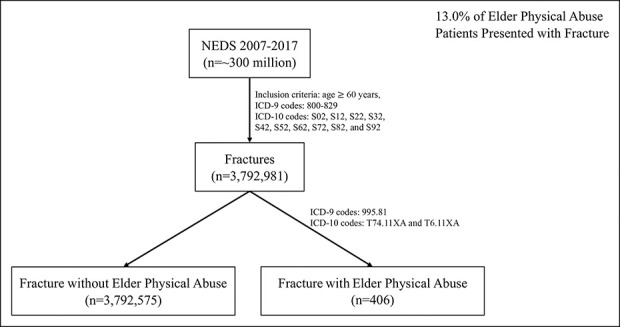
Flow diagram for data inclusion. Data were extracted from 2007 to 2017 National Emergency Department Sample (NEDS) using the abovementioned inclusion criteria.

All patients aged 60 years and older with an International Classification of Disease (ICD)-9 or ICD-10 (9th and 10th revisions) code of fracture were identified in the 2007 to 2017 National Emergency Department Sample database (Figure 1). The chosen age cutoff of 60 years used in this study is consistent with the Centers for Disease Control definition of elder abuse.^4^ Data were extrapolated to national estimates by applying a unique sample weighting factor provided for each discharge.

In addition, all patients with elder physical abuse (with or without fracture) were identified. These data were used to determine the percentage of elder physical abuse patients presenting with fracture.

### Patient Factors

Patient factors abstracted included age and sex. Income quartile was also abstracted from the data set. Furthermore, ICD codes allowed for characterization of comorbidities and injuries.

ICD-9 and ICD-10 diagnosis codes were used to identify patient characteristics, including osteoporosis, volume depletion, mental disorder, dementia, intellectual disability, other elder abuse (psychological, neglect, sexual, and unspecified/other), and fracture location (800-829, S02, S12, S22, S32, S42, S52, S62, S72, S82, and S92). Multiple fractures were defined as the presence of more than one diagnosis of fracture using ICD-9 and ICD-10 codes.

Adult physical abuse was identified based on the ICD-9 or ICD-10 diagnoses codes 995.81, T41.11XA, and T61.11XA. Patients were dichotomized into those with and without physical abuse.

### Statistical Analysis

Chi square tests and Student *t*-tests were used to compare baseline demographic factors, and multivariate logistic regression analysis was used to adjust for baseline differences in age, sex, and income quartile between groups. Missing variables were not included in the analysis.

All statistical analyses were conducted using STATA version 16 (StataCorp LP). Significance was set at *P* < 0.05.

## Results

### Study Population

From 2007 to 2017, 3,792,981 elder patients presented to EDs with fracture (Figure 1). Of these, 406 were identified as elder physical abuse patients (0.01%). Although this is a low percentage of patients presenting with fracture, it represents 13% of those presenting with elder physical abuse (406/3124). From 2007 to 2017, weighted national estimates of fracture was 17,054,672 with 1830 of those identified as elder physical abuse patients during that presentation.

### Univariate Analyses

Univariate analyses were initially conducted. Significant differences were identified in demographics between elder abuse and nonabuse patients presenting with fracture (Table 1). Elder abuse patients were found to be significantly younger (72 years versus 76 years, *P* < 0.001), be female (75% versus 69%, *P* = 0.003), and belong to lower income quartiles (*P* = 0.003) compared with their nonabuse counterparts.

**Table 1:** Demographic Factors in Elder Physical Abuse Fracture Patients Compared With Fracture Controls

	Elder Physical Abuse Fracture Patients (n = 406)	Fracture Controls (n = 3,792,575)	*P* Value
Age, mean ± SD, yr	72 ± 9	76 ± 10	**<0.001**
Female (%)	306 (75)	2,598,726 (69)	**0.003**
Income quartile^a^			**0.003**
First quartile ($1-$38,999)	109 (31.4%)	786,412 (25.4%)	
Second quartile ($39,000-$47,999)	90 (26.0%)	822,739 (26.6%)	
Third quartile ($48,000-$62,999)	92 (26.5%)	758,501 (24.5%)	
Fourth quartile ($63,000+)	56 (16.1%)	730,067 (23.6%)	

a Income quartiles are adjusted for each year; mid-point year income quartiles are provided.

Missing variables were not included in this analysis. Significance was defined at *P* < 0.05 and those meeting these criteria were in bold.

Clinical factors were also assessed for prevalence in elder physical abuse fracture cases (Table 2). The elder physical abuse group presented with more volume depletion (7.39% versus 3.43%, *P* < 0.001), mental disorders (53.2% versus 26.2%, *P* < 0.001), dementia (6.90% versus 3.41%, *P* < 0.001), and intellectual disability (^a^ % versus 0.18%, *P* < 0.001). Other forms of elder abuse—including psychological abuse, neglect, sexual abuse, and unspecified/other abuse—were also more prevalent in the elder physical abuse group (^a^% versus 0.01%, *P* < 0.001). Patients with osteoporosis were not markedly different between the two groups.

**Table 2:** Clinical Factors in Elder Physical Abuse Fracture Patients Compared With Fracture Controls

	Elder Physical Abuse Fracture Patients (n = 406)	Fracture Controls (n = 3,792,575)	*P* Value
Osteoporosis	36 (8.87%)	324,909 (8.57%)	0.83
Volume depletion	30 (7.39%)	130,226 (3.43%)	**<0.001**
Mental disorders	216 (53.2%)	995,279 (26.2%)	**<0.001**
Dementia	28 (6.90%)	129,305 (3.41%)	**<0.001**
Intellectual disability	^a^	6765 (0.18%)	**0.007**
Other elder abuse	^a^	275 (0.01%)	**<0.001**
Psychological	^a^	18 (0.00%)	**<0.001**
Neglect	^a^	185 (0.00%)	**<0.001**
Sexual	^a^	29 (0.00%)	**<0.001**
Unspecified/Other	^a^	178 (0.00%)	**<0.001**

a Indicates sample size ≤10.

Missing variables were not included in this analysis. Significance was defined at *P* < 0.05 and those meeting these criteria were in bold.

For fracture patterns, elder physical abuse patients, compared with their nonabuse counterparts, had a greater proportion of skull (23.2% versus 7.21%, *P* < 0.001) and rib (21.9% versus 12.5%, *P* < 0.001) fractures and fewer femur (13.1% versus 20.5%, *P* < 0.001) and foot/ankle (4.68% versus 11.1%, *P* < 0.001) fractures (Table 3). In addition, elder abuse patients were more likely to present with multiple fractures (20.2% versus 12.2%, *P* < 0.001).

**Table 3:** Incidence of Different Fracture Types in Elder Physical Abuse Patients

	Elder Physical Abuse Fracture Patients	Fracture Controls	*P* Value
Any fracture^a^	406	3,792,575	
Multiple fractures	82 (20.2%)	463,429 (12.2%)	**<0.001**
Skull	94 (23.2%)	273,370 (7.21%)	**<0.001**
Spine and pelvis			
Cervical vertebra	^b^	98,749 (2.60%)	0.62
Thoracic vertebra	16 (3.94%)	163,492 (4.31%)	0.71
Lumbar vertebra	30 (7.39%)	218,301 (5.76%)	0.16
Sacrum	^b^	53,450 (1.41%)	0.76
Pelvis	19 (4.68%)	215,202 (5.67%)	0.39
Rib/Sternum	89 (21.9%)	474,153 (12.5%)	**<0.001**
Rib	^b^	28,098 (0.74%)	0.25
Sternum			
Upper extremity			
Clavicle	13 (3.20%)	86,904 (2.29%)	0.22
Scapula	^b^	25,872 (0.68%)	0.46
Humerus	32 (7.88%)	334,630 (8.82%)	0.50
Radius/Ulna	46 (11.3%)	472,928 (12.5%)	0.49
Hand/Wrist	27 (6.65%)	274,163 (7.23%)	0.65
Lower extremity			
Femur	53 (13.1%)	779,167 (20.5%)	**<0.001**
Patella	^b^	63,289 (1.67%)	0.06
Tibia/Fibula	12 (2.96%)	124,623 (3.29%)	0.71
Foot/Ankle	19 (4.68%)	421,385 (11.1%)	**<0.001**

a Patients may present with multiple fractures in different locations.

b Indicates sample size ≤≤10.

Missing variables were not included in this analysis. Significance was defined at *P* < 0.05 and those meeting these criteria were in bold.

### Multivariate Analyses

Regarding clinical factors, elder physical abuse patients were more likely to present with volume depletion (2.21 [1.40 to 3.48]) and mental disorders (3.34 [2.70 to 4.12]), including dementia (2.86 [1.87 to 4.37]) and intellectual disability (3.89 [1.24 to 12.2]), even after adjusting for demographic factors (Table 4). Other forms of elder abuse were found to be markedly predictive of concurrent elder abuse: overall (262 [115-595]), psychological (2140 [687 to 6680]), neglect (369 [162 to 844]), sexual (250 [33.5 to 1870]), and unspecified/other (60.5 [8.43 to 435]). Osteoporosis was not predictive of elder abuse.

**Table 4:** Odds Ratios for Clinical Factors in Predicting Elder Physical Abuse

	Odds Ratio (CI)	Adjusted Odds Ratio (CI)	*P*
Volume depletion	2.24 (1.55-3.25)	2.21 (1.40-3.48)	**0.0007**
Mental disorder	3.19 (2.63-3.88)	3.34 (2.70-4.12)	**<0.0001**
Dementia	2.10 (1.43-3.08)	2.86 (1.87-4.37)	**<0.0001**
Intellectual disability	4.17 (1.34-13.0)	3.89 (1.24-12.2)	**0.02**
Other elder abuse	348 (184-660)	262 (115-595)	**<0.0001**
Psychological	3160 (1250-8000)	2140 (687-6680)	**<0.0001**
Neglect	465 (236-914)	369 (162-844)	**<0.0001**
Sexual	323 (43.9-2380)	250 (33.5-1870)	**<0.0001**
Unspecified/Other	52.6 (7.35-376)	60.5 (8.43-435)	**<0.0001**
Osteoporosis	1.04 (0.74-1.46)	1.15 (0.78-1.71)	**0.50**

Unadjusted odds ratios are provided (odds ratio) in addition to odds ratios adjusted for age group, sex, and income quartile (adjusted odds ratio)

Missing variables were not included in this analysis. Significance was defined at *P* < 0.05 and those meeting these criteria were in bold.

**Figure 2:**
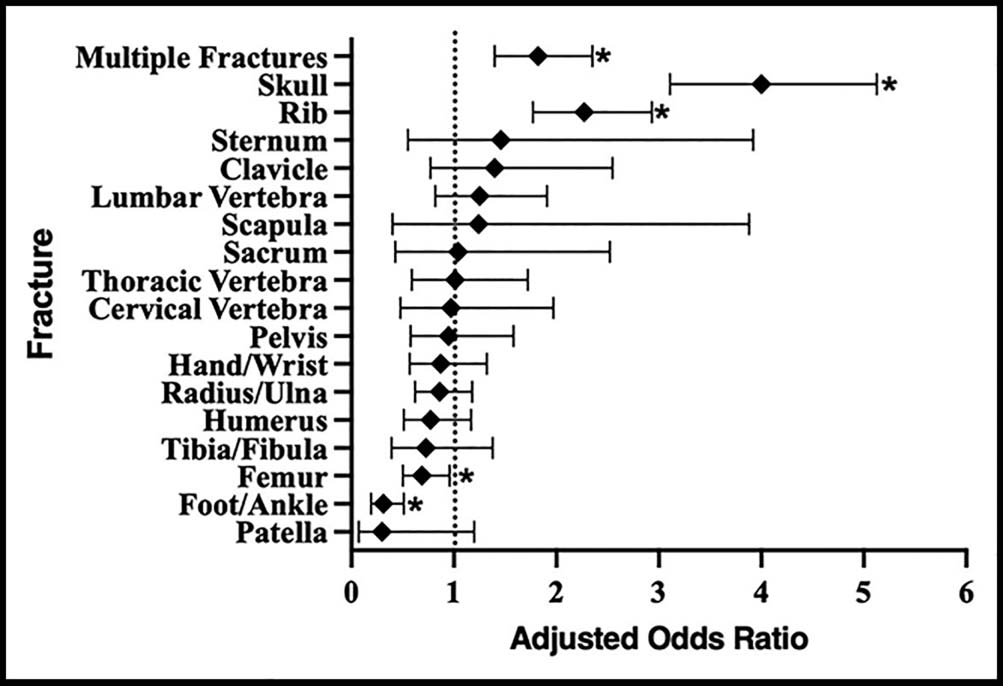
Graph showing odds ratios for fracture locations in predicting elder abuse. Odds ratios (◆) were adjusted for age group, sex, and income quartile with 95% CIs provided. The dotted line along odds ratio of 1.0 has been shown for reference. Significant odds ratios are demonstrated (*).

A separate regression was then used to determine whether fracture type could be helpful in identifying elder abuse (Table 5). Elder physical abuse patients were more likely to present with multiple fractures (adjusted odds ratio = 1.82 [95% CI = 1.40 to 2.35]). Skull (adjusted odds ratio = 4.00 [95% CI = 3.11 to 5.13]) and rib (2.27 [1.77 to 2.93]) fractures were found to have a higher odds ratio for predicting elder physical abuse while femur (0.69 [0.50 to 0.96]) and foot and ankle (0.31 [0.19 to 0.51]) fractures were found to be less likely because of elder physical abuse (Figure 2). No other fracture locations were markedly predictive of elder physical abuse.

**Table 5:** Odds Ratios for Fractures in Predicting Elder Abuse

	Odds Ratio (CI)	Adjusted Odds Ratio (CI)
Multiple fractures	1.82 (1.43-2.32)	1.82 (1.40-2.35)
Skull	3.89 (3.08-4.88)	4.00 (3.11-5.13)
Spine and pelvis		
Cervical	0.85 (0.44-1.64)	0.97 (0.48-1.97)
Thoracic	0.91 (0.55-1.50)	1.01 (0.59-1.72)
Lumbar	1.31 (0.90-1.90)	1.25 (0.82-1.91)
Sacrum	0.87 (0.36-2.11)	1.04 (0.43-2.52)
Pelvis	0.82 (0.52-1.29)	0.95 (0.58-1.58)
Rib/Sternum		
Rib	**1.96 (1.55-2.49)**	**2.27 (1.77-2.93)**
Sternum	1.67 (0.69-4.04)	1.46 (0.55-3.92)
Upper extremity		
Clavicle	1.41 (0.81-2.45)	1.40 (0.77-2.55)
Scapula	1.45 (0.54-3.88)	1.24 (0.40-3.88)
Humerus	0.88 (0.62-1.27)	0.77 (0.51-1.17)
Radius/Ulna	0.90 (0.66-1.22)	0.86 (0.62-1.18)
Hand/Wrist	0.91 (0.62-1.35)	0.87 (0.57-1.32)
Lower extremity		
Femur	**0.58 (0.44-0.78)**	**0.69 (0.50-0.96)**
Patella	0.29 (0.07-1.17)	0.30 (0.07-1.20)
Tibia/Fibula	0.90 (0.50-1.59)	0.73 (0.39-1.38)
Foot/Ankle	**0.39 (0.25-0.62)**	**0.31 (0.19-0.51)**

Unadjusted odds ratios are provided (odds ratio) in addition to odds ratios adjusted for age group, sex, and income quartile (adjusted odds ratio)

Missing variables were not included in this analysis. Significance was defined at *P* < 0.05 and those meeting these criteria were in bold.

### Example Application of Study Findings

**Figure 3:**
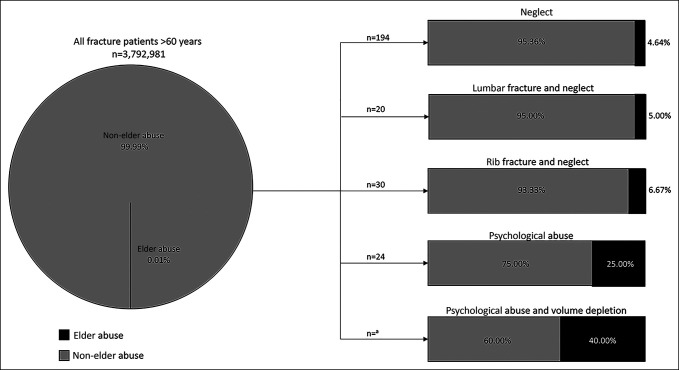
Diagram showing example clinical scenarios with higher proportions of elder physical abuse. The total incidence of elder abuse in the overall fracture population has been shown on the left. Elder abuse incidence in fracture patients presenting with other identifiable factors, including neglect, lumbar fracture with neglect, rib fracture with neglect, psychological abuse, and psychological abuse with volume depletion. ^a^ Indicates sample size ≤≤10.

Although the incidence of elder physical abuse was found to be low in those presenting with fracture (0.01% of those older than 60 years), if considering distinct clinical scenarios, the findings can amplify. Several specific scenarios are presented in Figure 3 to highlight this point.

Of those presenting with neglect, 4.64% would be expected to have elder physical abuse. Of those presenting with neglect and lumbar fracture, 5.00% would be expected to have elder physical abuse. Of those presenting with rib fractures and neglect, 6.67% would be expected to have elder physical abuse. Of those presenting with psychologic abuse, 25.0% would be expected to have elder abuse. Of those presenting with psychologic abuse and volume depletion, 40.0% would be expected to have elder abuse.

## Discussion

Identifying signs of physical abuse can be challenging for the orthopaedic surgeon, especially in the elderly who have a higher risk of fractures. Unfortunately, predictors of elder abuse, particularly involving fracture locations, have not been well defined to date. Thus, this study used a multiyear national ED sample of fracture patients to identify predictors for elder physical abuse and found that those with elder abuse were more likely to be younger; be female; belong to a lower income quartile; present with volume depletion, mental disorders, or other forms of elder abuse; have skull or rib fractures; and/or present with multiple fractures.

The fact that 13.0% of the elder physical abuse patients were found to present with fracture highlights that orthopaedic surgeons may be caring for such patients. As with pediatric patients, they should know which patients might need additional discussion/evaluation for physical elder abuse.

Of the population identified to have had elder physical abuse, a greater percentage were younger within the elderly cohort. This finding was also seen in another study analyzing elder abuse in Korea.^21^ It is unclear whether there is increased detection of elder abuse in younger patients and whether younger patients are at higher risk for elder abuse or for other reasons. The elder physical abuse cohort was also more likely to be female. Women may be at a greater risk for experiencing abuse as is seen in intimate partner violence,^22^ but results from previous studies in elder abuse have been mixed.^2,23^ Finally, those with elder abuse were more likely to be of a lower income quartile. It has been proposed that limited financial resources may be a stressor in the lives of patients and their caretakers, predisposing them to experiencing elder abuse.^24^ These demographic variables were controlled for in the subsequent multivariate adjusted analyses.

Fractures often do not present in isolation, and there may be other factors that orthopaedic surgeons find in patient presentation that may increase suspicion for abuse, such as the presence of multiple fractures. In addition, other forms of elder abuse were found to have very high odds ratios in predicting elder abuse. Previous studies support that a large proportion of abuse patients experience multiple forms of abuse.^6,13,25^ An important factor to note here would be that once elder abuse of any form is identified, a more extensive workup for abuse is triggered, resulting in an increased likelihood of detecting multiple forms of elder abuse in a single patient. Volume depletion may be a sign of neglect and was found to be associated with elder abuse. In addition, patients presenting with mental disorders, including dementia and intellectual disability, may also be associated with elder abuse. The findings discussed earlier are consistent with other existing literature on elder abuse.^6,13,25^

Subsequent analysis focused on fracture location. Fracture location may be a valuable factor in identifying patients who need additional workup for elder abuse. To assess whether fracture patterns could independently predict elder abuse, sex, age group, and income quartile were controlled for. This study found that skull and rib fractures may be seen more in elder abuse patients, whereas femur and foot/ankle fractures were less likely to predict elder abuse. Femur and ankle fractures have been associated with frailty in older patients who have had falls.^26^ Skull and ribs have been useful in predicting and diagnosing child abuse and may be useful in predicting elder abuse.^27-29^ Mechanisms of injury or fracture type should be explored in future studies, especially regarding skull and rib fractures.

Although this study benefits from a large, nationally representative sample of ED visits making it possible to detect rare diagnoses of elder abuse, this study has several limitations. The prevalence of diagnosed elder abuse in fracture patients older than 60 years according to this study was 0.001%. However, other studies have suggested that the actual prevalence is much higher. The reported incidence of elder abuse in this study may be an underestimate of the true incidence of elder abuse. Our analysis is limited to elder abuse diagnosed and documented through ICD codes, which is likely an underestimate of the true incidence of actual abuse. Because documentation and reporting of elder abuse is mandatory, we likely have a good estimate of confirmed elder abuse cases; however, this study is not able to assess those cases where elder abuse was not recognized by the treatment team at the point of diagnosis. Given this study used a large database, findings reaching statistical significance need to be explored further to assess for the clinical significance.

In summary, orthopaedic surgeons may be consulting on elder patients who are subjects of physical abuse and need to be cognizant of when this should be considered. Fractures of the skull and ribs, multiple fractures, volume depletion, mental disorders, and signs of other forms of elder abuse may all be helpful indicators to more accurately identify patients who could benefit from an additional workup for elder abuse. Clinical suspicion based on the other known predictors of abuse not studied in this article, such as inconsistencies within the history provided, should also be used to help define the need for investigation. This information adds meaningfully to the knowledge surrounding elder abuse and may be a helpful aid in diagnosis for clinicians in primary care, urgent care, ED, radiology, and orthopaedic surgery settings who are caring for elderly patients.
